# The Liquid Genioplasty: Different Techniques Compared

**DOI:** 10.1007/s00266-025-05557-6

**Published:** 2026-02-05

**Authors:** Mario Goisis, Sheila Veronese, Patricija Kasilovska, Olga Malakhova, Karim Hirache, Maria Maddalena Nicoletti, Andrea Sbarbati

**Affiliations:** 1 De Clinic, C.so di Porta Ticinese 68, 20123 Milan, Italy; 2 Department of Neuroscience, Biomedicine, and Movement, Section of Anatomy and Histology, University of Verona, P.le L.A. Scuro 10, 37134 Verona, Italy; 3 University of Campania Luigi Vanvitelli, Piazza Luigi Miraglia 2, 80138 Naples, Italy; 4 Maxilla Facial Department, 1 st November Hospital, Oran University, 31000 Oran, Algeria

**Keywords:** Liquid genioplasty, Filler, HA, Lipofilling, Dermgraft, Complications, Chin

## Abstract

**Background:**

Non-surgical genioplasty is one of the best choices in cases of chin retrusion, and it represents a solution for the aesthetic amelioration of facial profile. Recent concerns arose about the risk of bone resorption and vascular occlusion. Different procedures and products may lead to different results. This study reports the experience of a single facial plastic surgeon (M.G.) using non-surgical techniques to fill the chin.

**Methods:**

This retrospective study assesses 225 patients injected from January 2019 through March 2024 in private clinics in Milan (Italy), London (UK), and Dubai (UAE). Forty-five patients were injected with hyaluronic acid (Genefill DX 1x1 ml, BioScience GmbH, Dümmer, Germany), 90 underwent a lipofilling procedure, and 90 underwent a Dermgraft procedure, that is, a personalized lipofilling, modified by the main author. Half of the last 180 patients underwent shock waves before fat harvesting. Patients were followed up for 12 months, and facial angles and satisfaction data were analyzed. Three experts scored the results over time.

**Results:**

After all the treatments, facial angles were corrected, but the maintenance of the results varied for the different treatments. Notably, at 3 months, the levels of satisfaction were higher for the HA filler treatment, while at 1 year, the levels of satisfaction were very high for the Dermgraft.

**Conclusions:**

Liquid genioplasty is a safe technique to correct chin retrusion, with good results and high levels of patient satisfaction. The maintenance of the results varies according to the type of product injected.

**Level of Evidence V:**

This journal requires that authors assign a level of evidence to each article. For a full description of these Evidence-Based Medicine ratings, please refer to the Table of Contents or the online Instructions to Authors www.springer.com/00266.

## Introduction

Chin advancement genioplasty was first described by Otto Hofer in 1942 on a cadaver [1]. Genioplasty is designed to change the chin's position and/or shape to improve facial aesthetics. Surgical genioplasty can modify the genial area in all three planes. In this way, maxillofacial surgery can correct protrusive and asymmetric chins, narrow its posterior and anterior dimensions, and advance the recessive chin.

Numerous surgical techniques involving both intraoral and extraoral approaches have been developed for genioplasty. They include osteotomies and placement of bone grafts or implants (silicone, Gore-Tex, Medpor, porous block hydroxyapatite, acrylic, etc.) [2]. All these techniques involve the risks of major surgical complications, such as nerve damage, infections, and bone necrosis, and demonstrate some relapse and stability issues at long-term follow-up [3]. Moreover, mandibular bone resorption caused by the sub-periosteal placement of the chin implants has been reported [4].

In the last few years, the popularity of chin augmentation surgery has decreased. From 2015 to 2019, The Aesthetic Society reported a nearly 17% decline in chin augmentations, with a drop of 7% from 2018 to 2019 alone [5]. One of the reasons for this decrease is the increase in the popularity of liquid genioplasty, a minimally invasive surgical cosmetic procedure used to reshape and enhance the chin. This procedure was introduced over 40 years ago and consisted of a fat grafting at the chin level. More recently, hyaluronic acid (HA) injections have also started to be used for liquid genioplasty. This corresponds to the fact that, during the last ten years, HA filler has become one of the most common procedures in aesthetic medicine because of its fine-tuning capabilities and quick recovery time, making it an ideal choice to achieve natural-looking results with minimal downtime and a high level of patient satisfaction [6–12]. In a systematic review by Ou and colleagues, the authors observed that HA fillers are an effective and temporary solution for correcting chin retraction and enhancing chin augmentation, offering high patient satisfaction and a low risk of serious complications [13].

Some concerns about the safety of liquid genioplasty by HA were documented in 2018 and 2020 by Guo et al. [14, 15], who reported nine cases of HA-induced mental bone resorption, similar to the one previously described, correlated to surgical implants. To avoid these and other complications, choosing the right filler to inject is essential. Thus, a thorough understanding of HA’s rheological properties, such as viscosity, elasticity, and cohesiveness, is mandatory when using this technique [16].

This study presents the experience and results of a single clinician (M.G.) utilizing different surgical and non-surgical genioplasty techniques to correct chin retrusion in a large cohort of patients. It stands as one of the most extensive case series reporting outcomes for this specific deformity. Specifically, a comparison was made between the glabella-subnasale-pogonion angle pre- and post-treatment using HA, fat grafting, and the innovative Dermgraft procedure developed by the clinician. The aim was to objectively evaluate the results of these different treatment modalities. Additionally, patient satisfaction was assessed, and three cosmetic surgeons conducted blind evaluations of the outcomes.

## Materials and Methods

From January 2019 through March 2024, liquid genioplasty was conducted by a single facial plastic surgeon (M.G.) in private clinics in Milan (Italy), London (UK), and Dubai (UAE). The procedures were performed with the approval of these clinics. This is a retrospective study, which was conducted on patients’ charts in accordance with the 1964 Declaration of Helsinki and its later amendments. Informed consent was obtained to analyze and publish patient data and photographs.

### Eligibility Criteria for Treatment

To be eligible for the treatment, participants had to be at least 18 years old and exhibit evidence of chin retrusion, as indicated by a glabella-subnasale-pogonion angle of less than 164° for males and 166° for females.

Criteria of exclusion were: undergone dermal filler treatment (resorbable) in the chin, jaw, glabellar area or nose within 36 months before genioplasty; undergone fat injections or surgery or permanent filler in the chin or jaw before genioplasty; undergone any dental procedure during the study; undergone botulinum toxin injections in the upper lip within 6 months before genioplasty [12].

### Patients and Treatments

Over five years, 225 patients, with ages ranging from 20 to 73 years, were treated; 74% of these patients were between 21 and 50 years old. The patients considered for the present study underwent five types of treatment (as detailed below). To compare the results of the different procedures, an equal number of patients were selected for each procedure to form five groups. The selection was made by reviewing the medical records of patients treated during the study period. Records deemed eligible were included in the study and reviewed in reverse chronological order (from most recent to oldest). A total of 45 patients’ charts were selected for each group. In the first group, 45 patients received injections of HA filler (Genefill DX 1x1 ml, BioScience GmbH, Dümmer, Germany). The second group consisted of 90 patients who underwent lipofilling treatment using various fat processing techniques, including the Coleman technique, Lipocube (LipoCube, Kâğıthane/İstanbul, Turkey), and the Micrograft procedure (Seffi, Superficial Enhanced Fluid Fat Injection, Bologna, Italy). Half of these patients also received preventive shock wave (SW) treatment (described below) on the abdomen prior to fat harvesting. These patients were placed in a specific, separate group. The third group included 90 patients who received the Dermgraft and enriched Nanograft treatments (both by Go Easy SRL, Milan, Italy) (described below). Similar to the lipofilling group, half of these patients underwent preventive SW treatment in the abdomen before the Dermgraft and Nanograft procedures (the procedure was referred to as Dermgraft in the rest of the paper), and were assigned to a specific, distinct group.

For the study’s repeatability, it is essential to specify that the type of treatment received by all patients was completely independent of the subsequent analysis performed in this study. In any case, all patients received treatment according to their requests. All patients undergoing HA filler had expressly requested non-surgical treatment. All patients undergoing lipofilling had asked for a surgical solution because they were aware of the more long-lasting results. All patients undergoing lipofilling had previously been visited by the surgeon to verify their suitability for the procedure. Therefore, for instance, very thin patients were not treated. SW pre-treatment was proposed to all patients before lipofilling. Some patients chose not to undergo this additional treatment due to time constraints, as this pre-treatment had to be performed starting 3 weeks before fat harvesting. Fat, Dermgraft, and enriched Nanograft were harvested from the abdomen. Patient characteristics and all aspects of the treatment were thoroughly documented. The composition of the 5 groups was statistically compared, as described in the Analysis paragraph, to verify that the results of the treatments were not influenced by this composition.

### Face Evaluation

The evaluation of the patient before, during and after the treatment, and the treatment planning were based on a straightforward personal approach, the Multiple Angle Rational Injection’s Operational Planning (M.A.R.I.O). This approach is based on a Photometric analysis of the face. In particular, standardized photographs of the left face profile were taken. An iPhone 14 Pro max (Apple, Cupertino, USA) with a camera 12MP 2x Telephoto 48 mm,* f*/1.78 aperture was used to take the photographs without flash.

Subjects were asked to remove glasses or other accessories which may obstruct or alter the face profile. They were then asked to stand upright, with both arms relaxed and hanging at their sides, and look straight ahead with their lips relaxed. The head was maintained in a natural posture (NHP), and the left and right profiles were captured.

**Fig. 1:**
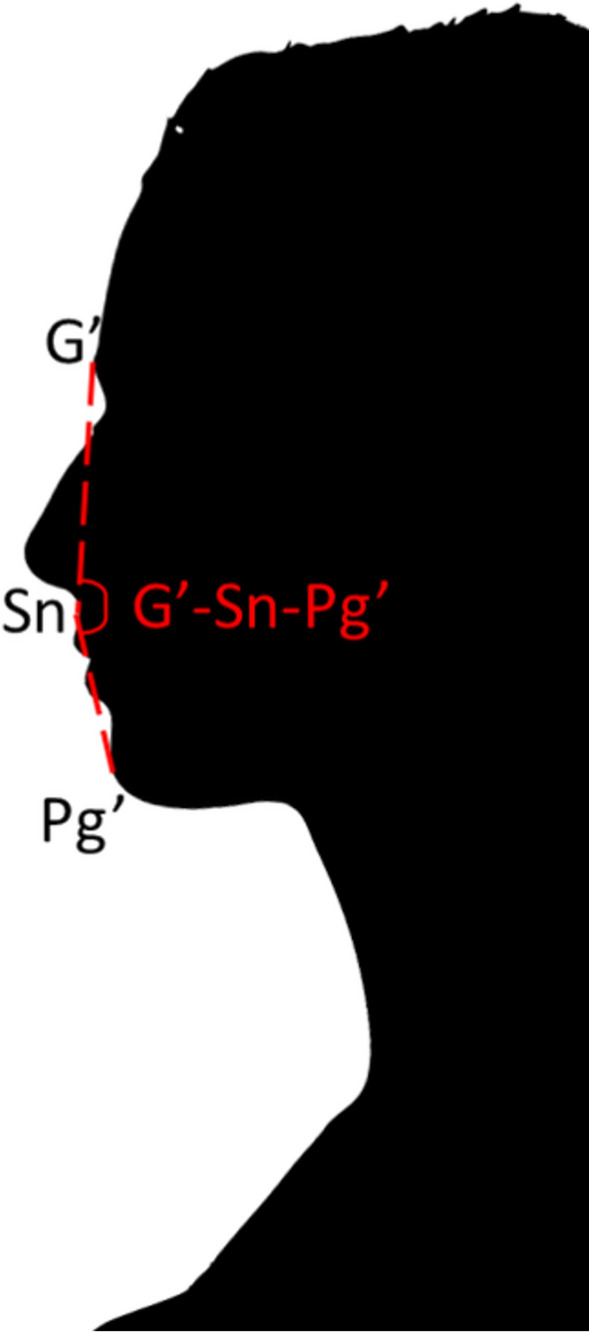
Schematization of the Glabella (G′), subnasale (Sn) and Pogonion (P’) points and of the G′–Sn–Pg′ angle

3 points were identified on the left profile of the patient (Fig. 1):

- G′ point or Glabella **(**or, better, cutaneous glabella)—the point in correspondence with the smooth elevation of the frontal bone just above the bridge of the nose;
- Sn point or Subnasale (or, better, cutaneous subnasale)—the point at which the nasal septum between the nostrils merges with the upper cutaneous tip in the midsagittal plane;
- Pg′ point or Pogonion (or, better, cutaneous pogonion)—the most anterior, prominent point on the chin.

The measurement of the facial angles was done using PowerPoint for Microsoft 365 (Microsoft, USA). The glabella was connected to the subnasale point with the ruler (Draw Tab—Tap the Ruler). Using the last point as a pivot, the ruler was rotated from the glabella to the pogonion and the rotation angle was measured. This angle corresponds to the facial convexity angle, or glabella-subnasale-pogonion angle (G′–Sn–Pg′). PowerPoint allows you to accurately measure angles in units of degrees. The illustrated technique had never been used in other studies, but the results obtained for the first 10 patients were aligned with those obtained by measuring the angles by hand or computer software. So, it was adopted for the remaining patients.

The PowerPoint slides were printed on Kodak glossy photo paper, size 15x21, using a Noritsu 1501 professional printer (Noritsu Koki Co Ltd, Tokyo, Japan) to guarantee a good definition and permit an optimal subsequent evaluation of the results.

The face pictures were acquired before and immediately after the treatment, and 3 and 12 months after. Subsequently, all the photographs were delivered to 3 blinded evaluators (3 plastic surgeons and aesthetic doctors) to assess the results. The evaluators were blinded to both the type of treatment and time points to obtain a completely objective evaluation of the results, not influenced by personal technical preferences, products, or instruments.

The retention rate of HA fillers is generally 9–12 months, according to the type of product. For this reason, the patients’ charts of the HA filler group were re-evaluated, and 27 patients treated with the HA filler were also evaluated 6 months post-treatment.

### Shock Wave Treatment

SWs were performed to improve the fat quality in the harvesting areas. The application on fat before the harvesting phase was pioneering, but the results have encouraged the authors to continue it, and, recently, it has been demonstrated that SWs produce beneficial effects on adipose tissue before fat grafting [17]. SWs were performed according to the Acoustic Wave Therapy (AWT) protocol (Storz Medical AG, Tägerwilen, Switzerland). AWT combines focused with radial acoustic waves. The therapy was performed twice a week, starting 3 weeks before fat harvesting. The application was performed in a 10 × 15 cm abdomen area, corresponding to the harvesting area.

The protocol consists of the application of a vibration therapy (2000 pulses of 3 bar at 50 Hz), followed by focused (1000 pulses at 1.24 mJ/mm^2^ at 8 Hz) and radial (4000 pulses at 5 bar at 21 Hz) SWs. All the procedures were applied using the DUOLITH® SD1 system with different probes, according to the type of stimulation to deliver (all devices from Storz Medical AG, Tägerwilen, Switzerland).

### Autologous Tissue Collection

First, 120 cc of Klein solution (2% Lidocaine solution: 0.08% *w*/*v*; Adrenaline 1 mg/ml solution: 0.1% *v*/*v* in 0.9% saline) were injected 10 min before the harvesting. A Goeasy cannula of 2 mm diameter, with 6 holes (Go Easy SRL, Milan, Italy) was used for the Dermgraft procedure. For the standard lipofilling procedure, the Coleman and the Seffi cannulas were used. The harvested tissue was processed as previously detailed.

Dermgraft differs from a standard lipofilling procedure as both superficial subcutaneous fat and deep dermal tissue are harvested and mixed. Nanograft is obtained by the emulsification and, consequently, disruption of the Dermgraft product. Finally, Enriched Nanograft results from the addition to Nanograft of the stromal vascular fraction components collected by the patented filtration system. As already stated, both Dermgraft and enriched Nanograft were reinjected.

### Treatment Administration

For lipofilling and Dermgraft, a Magic Needle with a 22G blunt-tip cannula (Needle Concept, Biarritz, France) was inserted at the level of the right and left modiolus. An average of 5cc of Dermgraft or fat was injected at the pogonion, mentum and sublabial crease level with a fanning technique.

For the filler, an average of 3cc of HA filler was injected in the same anatomical points as Dermgraft and fat. In order to reduce the risk of bone resorption in the long term, the surgeon avoided placing the HA in direct contact with the periosteum. The injection technique by a 22G cannula allows the placement of the HA in the hypodermis/subcutaneous layer.

In the present study, the HA filler was a suspension of positively charged dextranomer microspheres in a hydrogel of cross-linked hyaluronic acid sodium salt. This filler is not soluble in water, and it is produced from a hyaluronic acid that is not derived from animals but gained through fermentation. The gel presents longer retention than standard HA fillers thanks to the charged dextranomer that stimulates collagen production and its particular structure.

### Safety Assessments

The incidence of major complications was calculated to evaluate the safety of the treatments.

### Satisfaction

Patients were asked to fill out a questionnaire to evaluate the aesthetic results of the treatment. Their satisfaction level was scored using a 10-point Likert scale ranging from 1 to 10, where 1 corresponded to “dissatisfied” and 10 to “fully satisfied” with the correction.

The blinded evaluators had to answer a questionnaire, and for each photograph, there were 10 choices ranging from 1, corresponding to “much worse,” to 10, corresponding to “much improved.”

In particular, satisfaction was investigated immediately after the procedure and at 3 and 12 months of follow-up, both for patients and evaluators.

### Analysis

All the G′–Sn–Pg′ angles were normalized to an angle of 169°. The authors chose this angle in accordance with Fortes et al.[18] and Almeida et al. [19], who considered it a pleasant angle for Caucasian people, considering that the majority of the patients treated were of Caucasian origin (93%). Nevertheless, this angle may also be applicable to Saudi adults, in which a slightly greater convexity is reported [20] as a normal cephalometric norm. In fact, in this population, a mean difference of 2.93° ± 2.31° was reported. As a slight retrusion, as might be that of 169°, is generally considered pleasant, this choice appeared adequate. An explorative analysis of the G′–Sn–Pg′ angles and their variations was performed by a graphical representation of the different normalized measures of all the patients at the 4 different time points (before and immediately after the treatment, and at 3 months and 1 year of follow-up). For each group of patients, the minimum, maximum, mean, and standard deviation values were calculated and graphed. Due to the non-normal distribution of the data, nonparametric tests were conducted to detect group-wise differences. The significance level was set to 95% for all tests. The normalized data were analyzed using the Kruskal–Wallis and post-hoc Dunn tests to compare the groups. Bonferroni correction (*α *= 0.0002632) was applied. Differences over the time of a single treatment and among the various treatments at different time points were evaluated.

A qualitative analysis of the patients’ satisfaction levels and blinded evaluations of the treatments’ results were conducted by graphical representations of the scores of all the patients and experts at the 4 different time points. For each group of patients, the minimum, maximum, mean, and standard deviation values were calculated and graphed. Mean patients' satisfaction and experts’ evaluations were compared using the Mann–Whitney U test, with a significance level set to 95%. To detect differences among groups, the Kruskal–Wallis and post-hoc Dunn tests were used. Bonferroni correction (*α *= 0.00048) was applied. Differences over time and among the treatments were evaluated.

Statistics Kingdom 2017—web application (Statistics Kingdom, Melbourne, Australia) was used for all analyses, and Excel 2019 (Microsoft Corporation, Redmond, Washington, USA) was used to produce graphs.

## Results

### Patients and Treatment Results

Demographic data of the five different groups of patients are reported in Table 1, while the raw data of all patients’ G′–Sn–Pg′ angles, along with the mean and standard deviation, minimum and maximum values for each group, are presented in Table 2. These data and the statistical comparison of the 5 groups through the post hoc Dunn test for multiple analysis (with Bonferroni correction) revealed that the groups were statistically similar (groups were considered statistically different if *p *< 0.05; results: HA-lipofilling *p *= 0.9602; HA-lipofilling with SW *p *= 0.8748; HA-Dermgraft *p *= 0.8624; HA-Dermgraft with SW *p *= 0.9185; lipofilling-lipofilling with SW *p *= 0.8356; lipofilling-Dermgraft *p *= 0.8234; lipofilling-Dermgraft with SW *p *= 0.879; lipofilling with SW-Dermgraft *p *= 0.9875; lipofilling with SW-Dermgraft with SW *p *= 0.9559; Dermgraft-Dermgraft with SW *p *= 0.9434). This allows for comparing the results of the different treatments performed in the different groups of patients.

**Table 1:** Demographics

	Hyaluronic acid	Lipofilling	Lipofilling and shock waves	Dermgraft	Dermgraft and shock waves
Age range	22–58	21–62	19–57	23–73	20–59
Mean age	34	31	40	38	37
Females (%)	82	79	84	75	74

**Table 2:** Initial G′–Sn–Pg′ angles of all the patients and mean, standard deviation, minimum and maximum of the groups

Hyaluronic acid	Lipofilling	Lipofilling and shock waves	Dermgraft	Dermgraft and shock waves
*Initial G*′–*Sn–Pg*′ *angles of all patients*				
162	166	161	165	164
163	159	164	163	160
165	164	165	164	162
160	162	163	161	164
164	163	164	160	162
162	156	161	164	161
163	162	160	163	164
164	164	164	164	165
166	160	163	161	161
159	162	163	160	159
164	164	164	159	164
162	162	156	164	162
163	161	159	160	163
163	164	164	162	163
164	165	162	164	165
162	161	163	162	163
161	160	163	161	164
160	159	165	164	165
164	164	163	165	166
164	161	164	161	161
166	160	161	165	159
163	164	160	163	160
162	165	164	164	162
164	163	163	161	164
160	164	164	160	160
162	161	161	164	159
164	160	160	163	162
162	164	159	156	164
161	163	165	161	163
164	164	163	160	161
165	164	164	164	160
156	164	161	163	156
164	166	160	160	163
161	163	164	165	164
160	160	163	166	164
164	162	164	161	164
163	164	161	159	161
164	163	160	160	163
161	160	163	162	159
160	165	160	164	160
159	166	162	160	164
164	161	164	159	165
161	159	163	162	162
160	160	160	164	160
161	162	159	163	163
*mean±standard deviation*				
162.4 ± 2.1	162.4 ± 2.2	162.2 ± 2.0	162.1 ± 2.1	162.2 ± 2.1
*min*				
156	156	156	156	156
*max*				
166	166	165	166	166

**Fig. 2:**
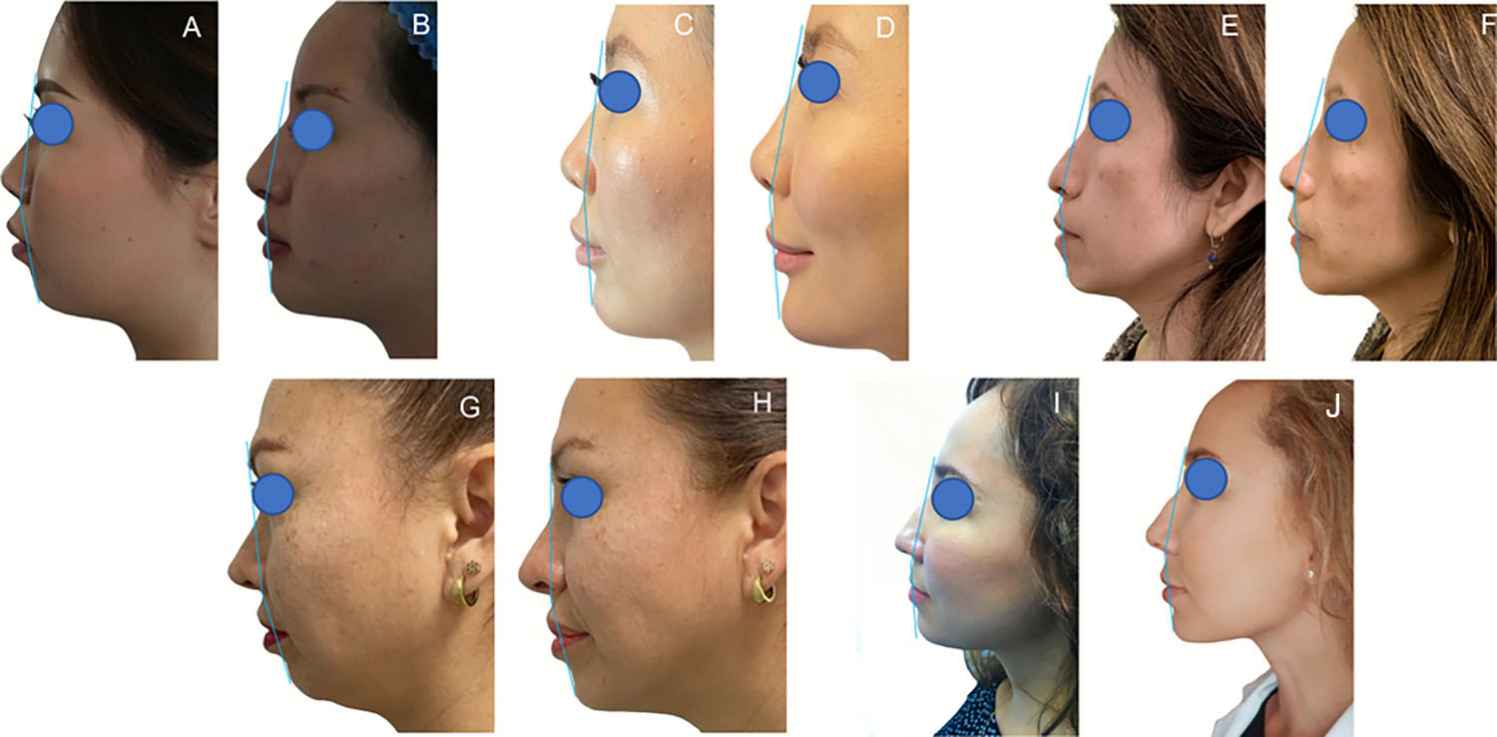
Results of the treatments. **A, B** Subject of the HA group, before and after 3 months from the injection of 3cc of HA filler. **C, D** Subject of the Lipofilling group, before and after 3 months from the lipofilling treatment. **E, F** Subject of the Lipofilling and shock waves group, before and after 3 months from the lipofilling treatment preceded by shock waves. **G, H** Subject of the Dermgraft group, before and after 3 months from the Dermgraft treatment. **I, J** Subject of the Dermgraft and shock waves group, before and after 3 months from the Dermgraft treatment preceded by shock waves

**Fig. 3:**
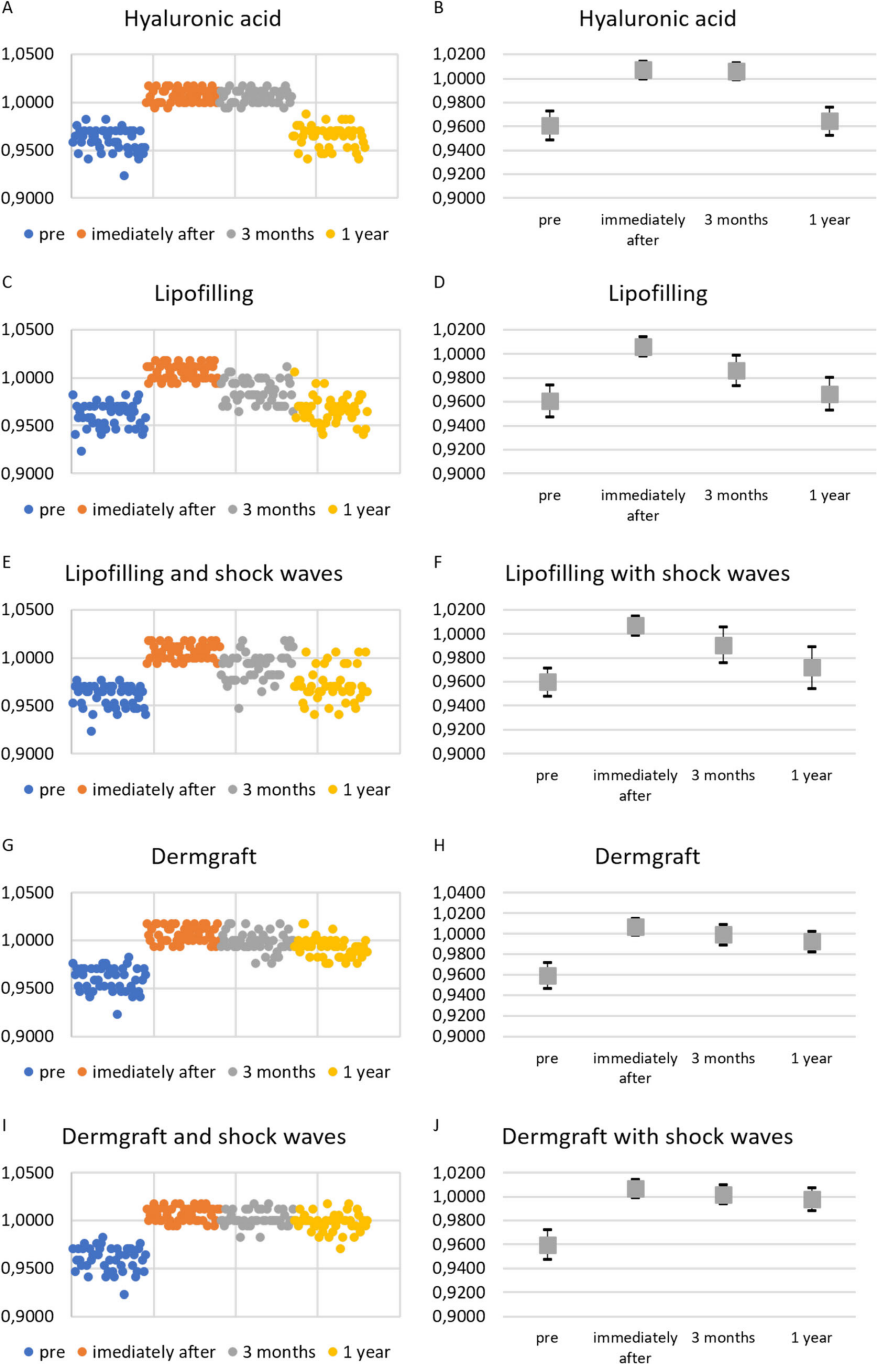
Results of the treatments over time. The single patients’ normalized angles were represented for all the treatments, and their mean and standard deviation were calculated and represented.** A** Normalized values of all patients’ G′–Sn–Pg′ angles at the different time points for HA filler treatment. **B** Mean and standard deviations of normalized values of all patients' G′–Sn–Pg′ angles at the different time points for HA filler treatment. **C** Normalized values of G′–Sn–Pg′ angles of all the patients treated with lipofilling at the different time points. **D** Mean and standard deviations of normalized values of G′–Sn–Pg′ angles of all the patients treated with lipofilling at the different time points. **E** Normalized values of G′–Sn–Pg′ angles of all the patients treated with lipofilling with SW at the different time points. **F** Mean and standard deviations of normalized values of G′–Sn–Pg′ angles of all the patients treated with lipofilling with SW at the different time points. **G** Normalized values of G′–Sn–Pg′ angles of all the patients treated with Dermgraft at the different time points. **H** Mean and standard deviations of normalized values of G′–Sn–Pg′ angles of all the patients treated with Dermgraft at the different time points. **I** Normalized values of G′–Sn–Pg′ angles of all the patients treated with Dermgraft with SW at the different times. **J** Mean and standard deviations of normalized values of G′–Sn–Pg′ angles of all the patients treated with Dermgraft with SW at the different time points

Immediately after all the treatments, all patients’ G′–Sn–Pg′ angles were corrected (Figs. 2 and 3).

It was found that the duration of persistence of effects differs between treatments (Fig. 3). At 3 months of follow-up, only the HA filler maintained the effect on the angle (the difference between the angles measured immediately after the treatment and at 3 months of follow-up was not statistically significant, *p *= 0.7685). All the other treatments presented an effect decay. This decay was statistically significant only for the lipofilling and the lipofilling with SW treatments (*p *< 0.0001). Subsequently, from the third month to 1 year of follow-up, a statistically significant decay was observed for all the treatments (*p *< 0.0001) except for Dermgraft and Dermgraft with SW.

**Fig. 4:**
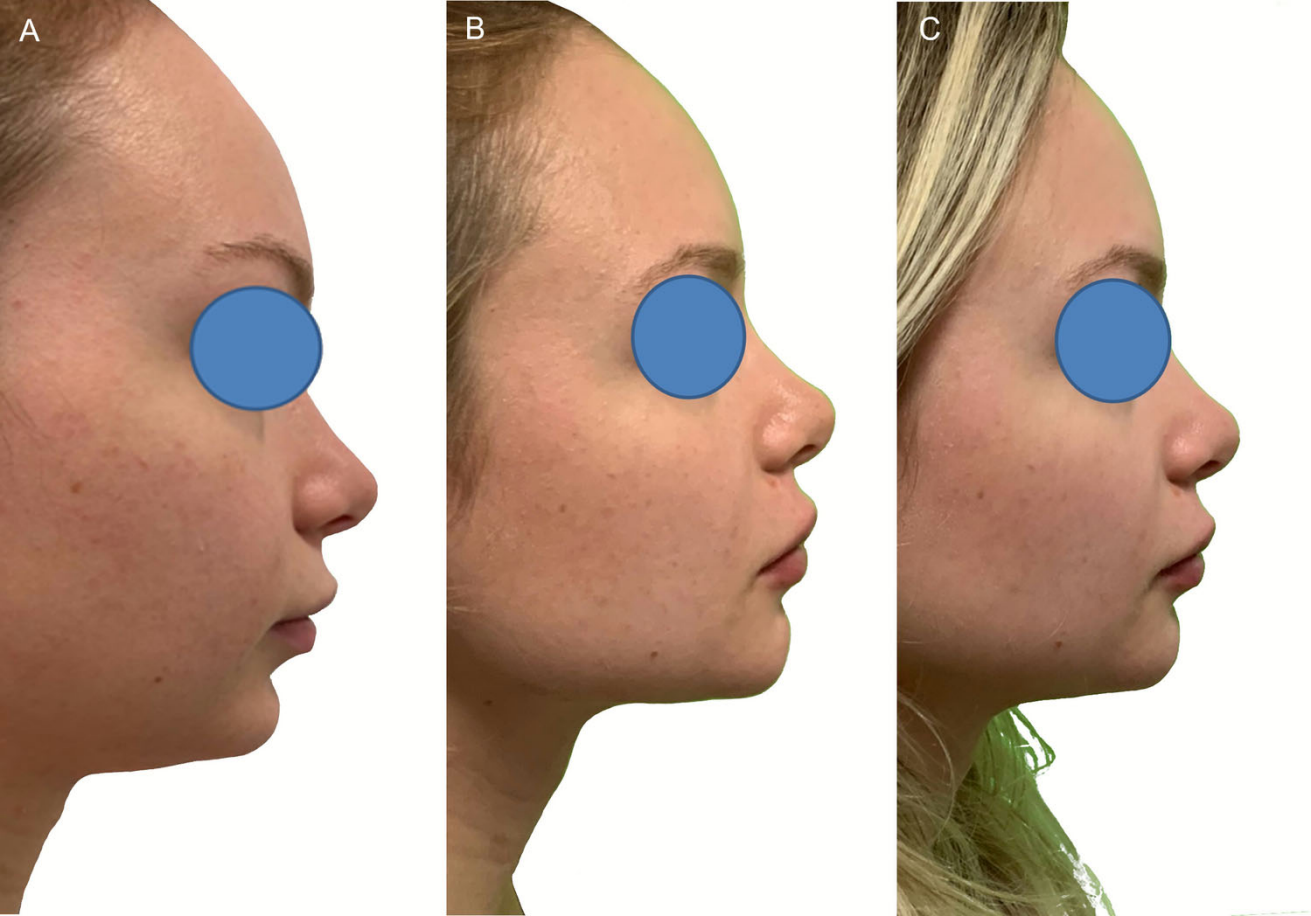
Results of the treatments. **A, B** One of the subjects before and after 3 months from the injection of 3cc of HA filler. **C** At 6 months of follow-up, there still was a partial maintenance of the corrective effect

Overall, the decay from the treatment (immediately after) to 1 year of follow-up was statistically significant in all the treatments (*p *< 0.0001) except Dermgraft with SW (the difference between the post-treatment angles and the ones measured at 1 year of follow-up was statistically significant, *p *= 0.01276). It must be said that the additional evaluation of the 27 patients treated with HA filler, at 6 months of follow-up, demonstrated a good maintenance of the effects (Fig. 4). Thus, the decay of the effects occurs in the six following months.

**Fig. 5:**
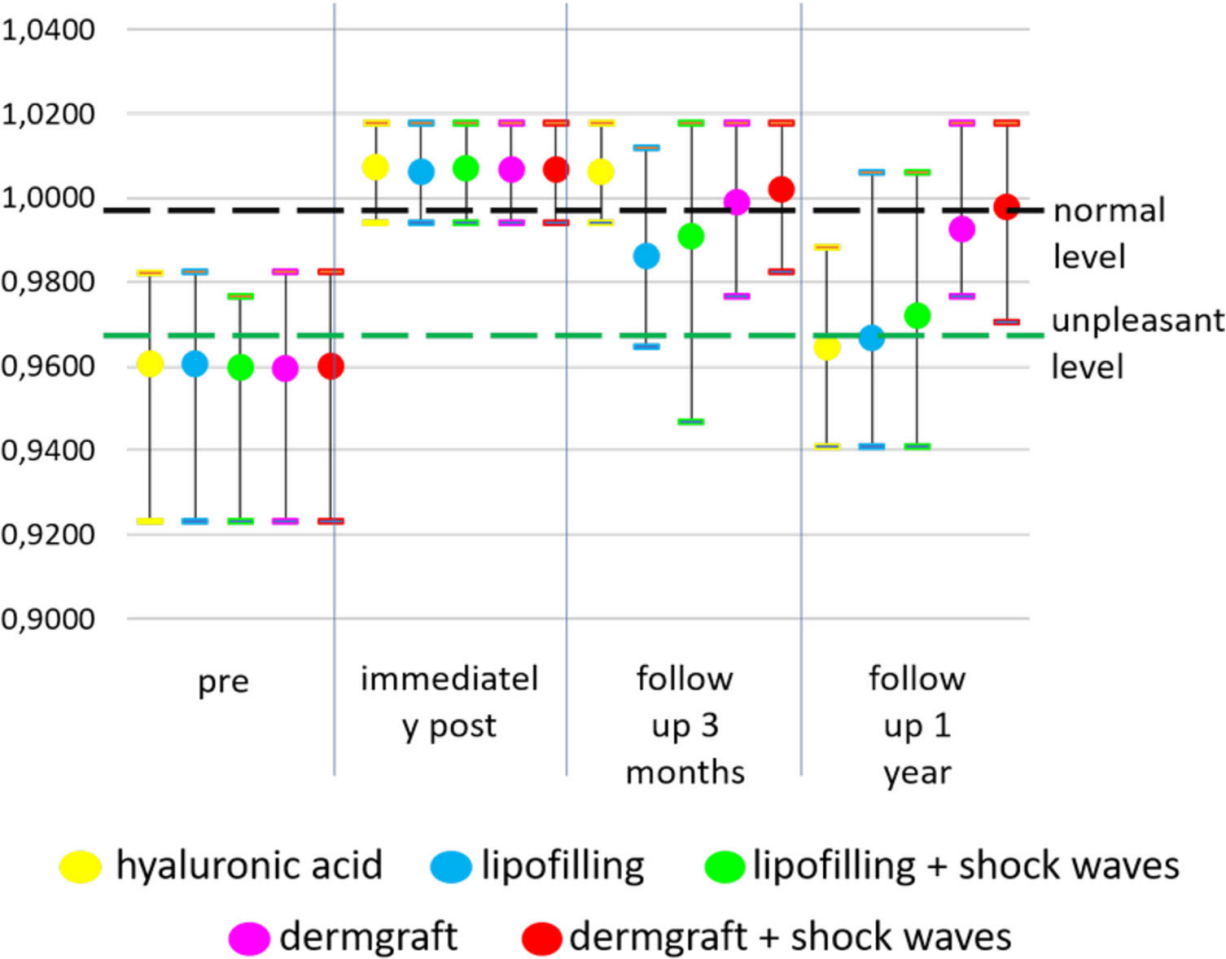
Maximum, minimum and mean of normalized values of G′–Sn–Pg′ angles of all the patients treated with the different procedures at the different time points

Comparing the G′–Sn–Pg′ angles before the treatments and at 1 year of follow-up, there were no statistical differences among the patients who had undergone HA filler, lipofilling and lipofilling with SW treatments. As a result, it is possible to state that these corrections were not maintained. Nevertheless, the improvements remained highly significant (*p *< 0.0001) for patients who received the Dermgraft and Dermgraft with SW treatments (Fig. 5).

### Safety Evaluation

The most commonly reported complications were firmness, redness, and pain after injection. No major complications, such as skin necrosis, embolism or infection, were reported.

In particular, the incidence of minor complications was lower after injection of HA filler (5%) and Dermgraft (6%) compared with the standard lipofilling technique (11%)

### Patients’ Satisfaction and Experts’ Evaluation

**Fig. 6:**
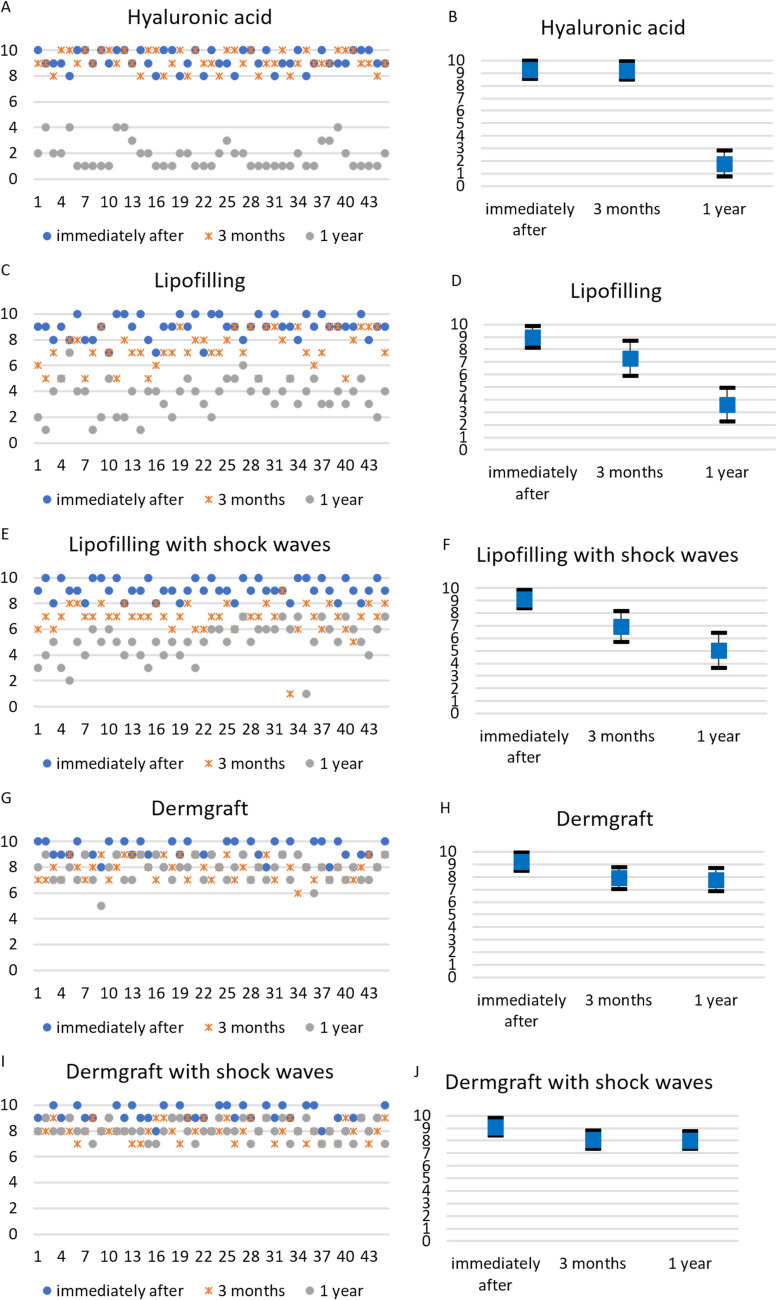
Patients’ satisfaction levels. The patients’ satisfaction levels were represented for all the treatments, and the mean and standard deviation were calculated and represented. **A** Satisfaction levels of all the patients injected with HA filler at the different time points. **B** Mean and standard deviation values of the satisfaction of all the patients injected with HA filler at the different time points. **C** Satisfaction levels of all the patients treated with lipofilling at the different time points. **D** Mean and standard deviation values of the satisfaction of all the patients treated with lipofilling at the different time points. **E** Satisfaction levels of all the patients treated with lipofilling with SW at the different time points. **F** Mean and standard deviation values of the satisfaction of all the patients treated with lipofilling with SW at the different time points. **G** Satisfaction levels of all the patients treated with Dermgraft at the different time points. **H** Mean and standard deviation values of the satisfaction of all the patients treated with Dermgraft at the different time points. **I** Satisfaction levels of all the patients treated with Dermgraft with SW at the different time points. **J** Mean and standard deviation values of the satisfaction of all the patients treated with Dermgraft with SW at the different time points

The patients’ satisfaction level and the experts’ evaluation scores reflect the results of the different treatments and their decay over time. Patients decreased their level of satisfaction from an initial good/optimal level for all the treatments to a value that follows the decay (Fig. 6).

At 3 months of follow-up, the patients’ satisfaction levels were higher for the HA filler treatment and lower for all the other treatments. For HA filler treatment, the satisfaction evaluation immediately after the treatment and at 3 months of follow-up was similar (non-significant difference, *p *= 0.8666), signifying a good maintenance of the noticeable effect. For all the other treatments, a statistically significant difference was noticed among the evaluations immediately after the treatments and at 3 months of follow-up (*p *< 0.0001), which means a noticeable reduction in the correction effect. Consequently, it is possible to state that the results obtained with the HA filler were statistically more satisfying with respect to the results obtained with the other treatments (*p *< 0.0001).

At the 1-year follow-up, satisfaction levels remained very high for the Dermgraft and Dermgraft with SW treatments, reflecting the sustained effects of these treatments. The satisfaction levels evaluated at 3 months and 1 year of follow-up did not significantly change (non-significant difference: *p *= 0.7146 for Dermgraft; *p *= 0.9276 for Dermgraft with SW). HA filler and lipofilling treatments showed a lower level of satisfaction in comparison to Dermgraft treatments, with a statistically significant reduction (*p *= 0 for HA filler; *p *= 0 for lipofilling; *p *= 0.009576 for lipofilling with SW).

**Fig. 7:**
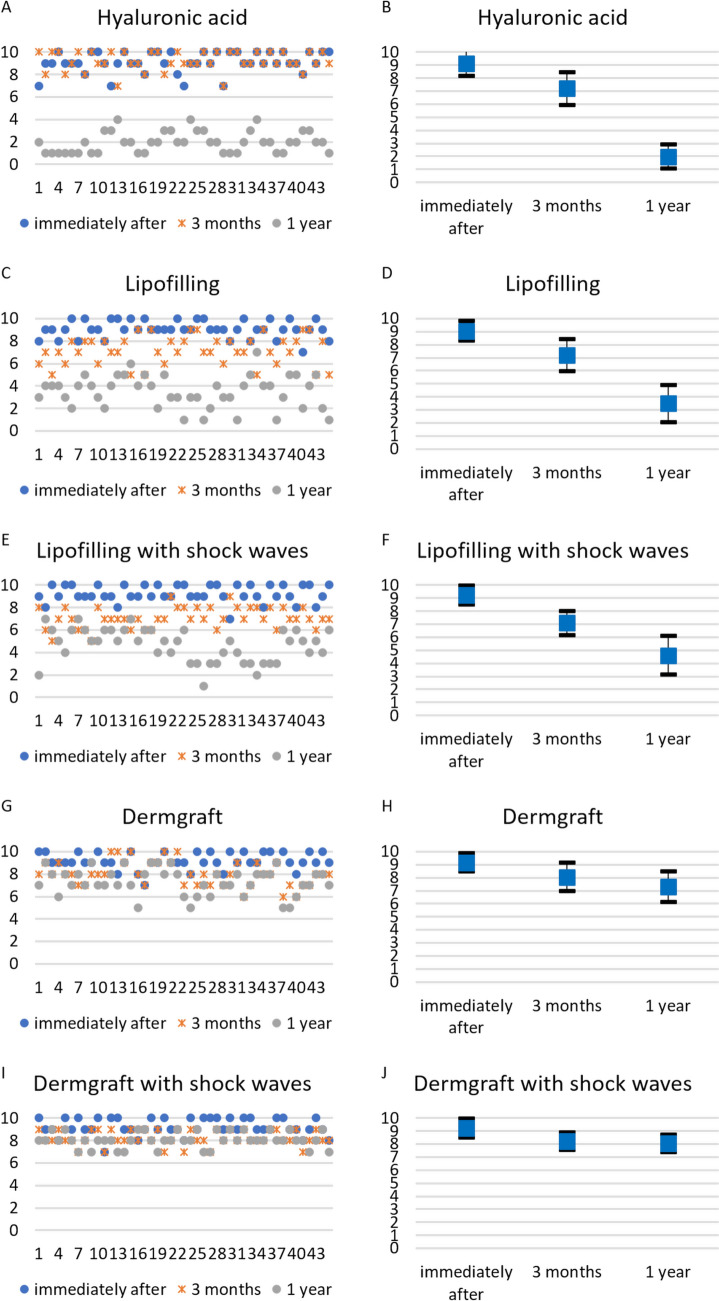
Experts’ evaluations. The experts’ evaluation score for each patient was represented for all the treatments, and the mean and standard deviation of these scores were calculated and represented. **A** Score for all the patients injected with HA filler at the different time points. **B** Mean and standard deviation values of the score for all the patients injected with HA filler at the different time points. **C** Score for all the patients treated with lipofilling at the different time points. **D** Mean and standard deviation values of the score for all the patients treated with lipofilling at the different time points. **E** Score for all the patients treated with lipofilling with SW at the different time points. **F** Mean and standard deviation values of the score for all the patients treated with lipofilling with SW at the different time points. **G** Score for all the patients treated with Dermgraft at the different time points. **H** Mean and standard deviation values of the score for all the patients treated with Dermgraft at the different time points. **I** Score for all the patients treated with Dermgraft with SW at the different time points. **J** Mean and standard deviation values of the score for all the patients treated with Dermgraft with SW at the different time points

Consistent with the trend in patient satisfaction, the experts’ evaluations were high immediately after treatment but declined as the effects diminished over time. At the 3-month follow-up, high evaluation scores were maintained only for the HA filler treatment. There were no statistically significant differences between the experts’ evaluations immediately after the HA filler treatment and at 3 months of follow-up (*p *= 0.78), signifying the sustained effects over time. For the other treatments, the effects at the 3-month follow-up were statistically reduced (*p *< 0.001). However, by the 1-year follow-up, the evaluations for the HA filler had significantly decreased (*p *= 0), while they remained high for the Dermgraft and Dermgraft with SW treatments. For these two treatments, maintenance of the effects was noted (differences in experts’ evaluation between 3 months and 1 year of follow-up were non-statistically significant: *p *= 0.05406 for Dermgraft; *p *= 0.536 for Dermgraft with SW) (Fig. 7).

**Fig. 8:**
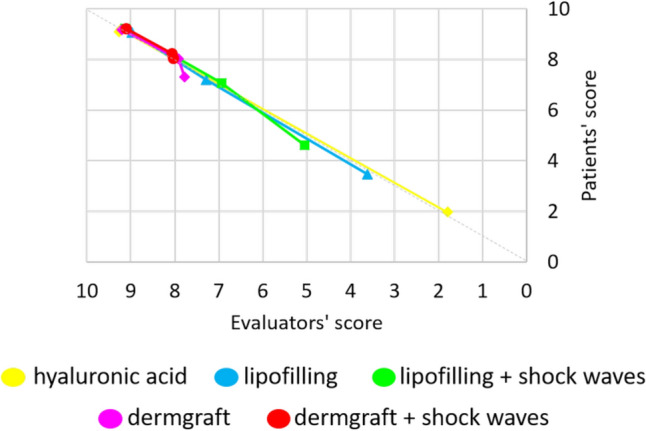
Comparison of expert ratings and patient satisfaction levels. The almost linear trend along the median indicates that the 2 ratings are consistent, i.e., the patients’ satisfaction is similar to the experts’ rating. The decreasing trend is related to the decrease in the effectiveness of treatments over time

The groups of the mean patients’ satisfaction and experts’ evaluation scores, compared using the Mann–Whitney U test, were statistically similar (the hypothesis that the two groups were different was rejected, *p *= 0.917) (Fig. 8).

## Discussion

One of the main goals of aesthetic treatments is to obtain a harmonious facial profile. The interest in facial contours has brought about decades of investigations of many gender and ethnic groups. Most of these aimed to define specific standards by cephalometric analysis (lateral cephalometric radiograph).

Riedel et al. [20] stated that the soft‐tissue profile and the underlying skeletal profile of the patient were closely related. Analogously, in their studies, Mauchamp et al. [21] and Barnett [22] highlighted how all soft‐tissue alterations were associated with skeletal alterations. It appears clear that an accurate face analysis is fundamental before every facial aesthetic procedure. For this reason, standardized photographs have gained significant clinical and research importance mainly because they reproduce the soft tissues in detail and are easy to perform [23]. Facial images are also helpful in evaluating patients' aesthetic expectations and demonstrating results, as done in the present study.

One of the standard parameters used to evaluate the intermaxillary relationships is the G′–Sn–Pg′ angle, which can indicate maxillary and mandibular misrelation in the sagittal plane. In this respect, significant differences have been identified in cephalometric norms relating to different populations, such as the Japanese [24], Chinese, Malay, Indian [25], Australian aborigines [26], Swedish [27], Africans, and Iranians [28, 29].

These publications and the present study’s data highlight a strong association between facial profile esthetics and the G′–Sn–Pg′ angle. Chin retrusion decreases the harmony of the face in the middle and lower thirds, and profiles in which this angle is reduced are considered less aesthetically pleasant [18, 23, 30]. A normal chin projection angle is, in general, preferred among people and considered aesthetically better than both protrusive and retrusive morphologies. In this regard, the study of Choi et al. [30] is of particular interest. They classified the preferred chin projection (CP) in the Korean population. 3D facial images were acquired using a 3D facial scanner. In each 3D facial image, normal CP was set to 10° (170° for the G′–Sn–Pg′ angle). Then, CPs were morphologically classified from normal to moderately protrusive (CP=6°, G′–Sn–Pg′=174°), slightly protrusive (CP=8°, G′–Sn–Pg′=172°), slightly retrusive (CP=12°, G′–Sn–Pg′=168°), and moderately retrusive (CP=14°, G′–Sn–Pg′=166°). Seventy-five dental students scored the CPs (6°, 8°, 10°, 12°, and 14°) from the most to least preferred. The normal chin projection angle was the most preferred without any difference between males and females in the mesoprosopic face.

The discussion about which angle to consider appropriate in terms of pleasant and unpleasant is still open. Fortes et al. [18] and Almeida et al. [19] reported that the mean value for this angle was 168.8 0 ± 4.44° in pleasant males and 169.60 ± 3.33° in pleasant females of the European (Caucasian) population. The mean value in unpleasant males was 165.40 ± 6.56°, and 164.93 ± 5.19° in unpleasant females. This highlights how unpleasant a chin retrusion is in both sexes. Similarly, Ogilvie et al. [12] affirmed that a chin retrusion should be corrected in subjects with G′–Sn–Pg′ facial angle inferior to 165°.

In the present study, patients presenting a skin retraction asked for a correction of the G′–Sn–Pg′ angle. An average increase of 7.9° in the G′–Sn–Pg′ angle was observed immediately after injecting all the products, followed by a progressive decrease over time. The decrease rate was different for the different treatments.

It is interesting to note how the results of HA filler treatment were utterly stable for the first 3 months, with partial resorption at 6 months and quite complete resorption of the product at 12 months. Instead, the other treatments presented a progressive decrease of the G′–Sn–Pg′ angles, particularly fast for the lipofilling and lipofilling with SW treatments.

The organism’s adaptation to the injected product could justify an initial decrease. Subsequent decreases induce the authors to think the treatments are unsuitable for retrusive problems or may not be ideal long-term solutions. The maintenance of the effects after Dermgraft and Dermgraft with SW treatments seems ideal for long-term solutions, and it is probably correlated to the type of product injected. Firstly, the procedure involves grafting superficial fat, which is less fibrotic than deeper fat layers, and may be more suitable for this application. Secondly, the Dermgraft processing system appears to preserve and activate adipose-derived stem cells (ADSCs), which are present in high quantities within adipose tissue [31–33]. Analogous results were found in a previous study, in which 4 samples processed by the Coleman technique, 4 by Lipocube system/Micrograft, 4 by Lipocube system/Nanofat, 4 by Seffi, 4 by Goeasy.bio system (Dermgraft) were analized. The cell growth results were higher for the stem cells obtained from Dermgraft compared to the others [34].

The advantage of adipose tissue use lies in the fact that it avoids any possible side effects concerning bone contact. Fat grafting has already been demonstrated to be efficient in bone regeneration. Thanks to the ADSCs’ properties, it has been successfully applied to repair bone defects stably and durably [35, 36]. It is mandatory to specify that there are many processing techniques/systems on the market [37–39] that produce different effects on ADSCs. Consequently, one method might be more effective than another in ADSCs activation, and this is also strongly related to the type of subsequent application.

A crucial data point emerging from this study is how both lipofilling and Dermgraft presented long-lasting effects if SW were previously applied. The differences between lipofilling and lipofilling with SW treatments and between Dermgraft and Dermgraft with SW treatments were not statistically significant, but the trend is evident. Perhaps the optimization of these combined treatments has yet to be reached.

More considerations may be made related to the HA use. It is the opinion of the authors that the injection of HA in the chin area requires particular attention. Guo et al. [40] conducted a prospective, controlled, observer-blind clinical trial to evaluate the severity of bone erosion after a single-shot injection in the symphyseal area at the periosteal level. The incidence of resorption was high, and the median bone resorption thickness ratio was 24.08%. No lesions, such as root apices injuries or mandibular symphyseal fractures, were found during the follow-up. Interestingly, the average bone resorption in that study was more significant in the subgroup of patients injected with more than 1 ml of HA. For this reason, a radiographic examination was suggested after about 3 years of serial injection of HA in the mentum. Thus, it is crucial to take into consideration the fact that the use of a blunt cannula allows for avoiding touching the periosteum and the direct contact between the bone and the HA. The personal authors’ experience confirms the safety of this technique, and, in this study, no cases of bone resorption or decrease of the facial angle were observed 12 months after the injection.

Excluding bone erosion, in accordance with Guo et al.[40], in the present study, no major side effects were reported correlated with HA injections. Among the worrisome complications of liquid genioplasty are skin necrosis and blindness due to vascular embolisms. In 2020, Lee et al. [41] reviewed 50 reports of filler-induced visual impairment, but they did not report cases of blindness linked to chin injections. Conversely, different case series of chin skin infarction and tongue necrosis after chin augmentation with HA have been reported [42–44]. These complications might be related to intravascular injection in the dominant ascending mental artery, and strengthened the necessity to use a blunt cannula in these corrections.

A last consideration must be made related to the type of HA filler injected. The HA filler used in this study is a hybrid product, and its specific characteristics make it ideal to warrant a good product integration in the injection site with a larger retention rate than other HA fillers. Moreover, the composition of this product may justify the relatively low percentage of minor complications, which was observed in this study and a previous study by Ossana et al. [34] in which the same product was off-label injected in the nose area. The choice of the HA filler must always be well calibrated.

Concerning the evaluations of the results, the data were fascinating, considering how patients’ satisfaction was aligned with experts’ evaluations. This means that the effects, maintenance, and decay of the treatments were so evident that patients’ and experts’ responses were coherent. It might be interesting to understand if the costs of the different procedures partly influenced the patients’ answers.

Finally, some concerns may arise from the fact that a single surgeon performed the treatments. Two possible points of view may be discussed. On the one hand, this may be seen as a limitation because other surgeons did not try the different procedures, and, consequently, it is impossible to generalize this study's findings. Conversely, a single surgeon could be perceived as an advantage that prevents bias correlated to different expertise and ability.

## Conclusions

All the possible risks correlated to the peculiar face anatomy of the chin lead to the consideration that, before intervening in this area and on the face, in general, it is crucial to do a deep anatomical analysis of the site. The results obtained in the present study demonstrated that the liquid genioplasty technique is relatively not dangerous and may be easily performed by using a blunt 22G cannula.

The HA filler treatment demonstrated an effect of less than one year. Despite this, it remains an effective option for patients seeking temporary and adjustable results with an average duration of 6–9 months. Moreover, it is a non-surgical procedure, which may be better tolerated and accepted by some patients. In contrast, the Dermgraft technique provided more durable outcomes, making it a preferable choice for those desiring longer-term corrections.

These findings highlight the versatility of HA-based treatments and underscore the importance of selecting the appropriate approach based on individual patient needs and expectations.

Finally, for those patients who choose to undergo a lipofilling, using a preventive SW fat treatment before the grafting could be the additional ingredient to trigger the activation of the ADSCs. However, the optimization of the parameters still needs to be achieved.
